# Cross-sectional study of myopia prevalence and associated risk factors among children and adolescents in Shaanxi Province, China, in 2021

**DOI:** 10.1080/07853890.2025.2522319

**Published:** 2025-06-22

**Authors:** Yi-Ming Guo, Guan-Chen Liu, Junhan Wei, Jiaqi Wang, Jiejing Bi, Juan Huang, Dang-Xia Zhou, Lu Ye

**Affiliations:** a Shaanxi Eye Hospital, Xi’an People’s Hospital (Xi’an Fourth Hospital), Affiliated People’s Hospital of Northwest University, Xi’an, China; b School of Life Science and Technology, Xi’an Jiaotong University, Xi’an, China; c Department of Pathology, School of Basic Medical Sciences, Health Science Center, Xi’an Jiaotong University, Xi’an, China

**Keywords:** Myopia, risk factors, children, adolescents, Shaanxi Province, epidemiology

## Abstract

**Purpose:**

Myopia is a growing global health issue, particularly among Chinese children and adolescents. This study aimed to investigate the prevalence and risk factors of myopia among children and adolescents in Shaanxi Province, China.

**Methods:**

A cross-sectional study conducted in 2021 included 261,504 participants from Guanzhong, Southern Shaanxi, and Northern Shaanxi. Ophthalmological examinations were performed, and refractive error was assessed using non-cycloplegic refraction to determine the spherical equivalent (SE). Myopia was defined as SE ≤−0.5 D and categorized into low (SE >−3.0 D), moderate (SE >−6.0 D), and high myopia (SE ≤−6.0 D). Data on age, gender, education level, and ethnicity were collected using structured questionnaires administered through face-to-face interviews.

**Results:**

The overall prevalence of myopia was 67.4% (95% CI: 67.20%–67.50%), with high myopia at 4.63% (95% CI: 4.55%–4.71%). Myopia was more common in females and increased with educational level, reaching 92.48% in senior high school students. Northern Shaanxi exhibited the highest prevalence of both myopia and high myopia. Regression analysis identified gender, education level, and region as significant risk factors.

**Conclusions:**

Myopia is highly prevalent among children and adolescents in Shaanxi Province, with notable gender, educational, and regional disparities. The findings underscore the urgent need for region-specific and education-level-targeted myopia prevention strategies.

## Introduction

Myopia is a type of refractive error where the eye’s refractive power causes light to focus in front of the retina, resulting in blurred distance vision [1]. The number of individuals with myopia has been increasing annually, with global prevalence expected to reach 49.8% by 2050, and high myopia rising from 2.7% to 9.8% [2]. The prevalence of myopia varies significantly across different regions, countries, and ethnic groups. In Africa and South America, myopia rates are below 10% among children, while North America shows a prevalence of approximately 42% [3]. In Europe, myopia rates differ considerably; for instance, a study in Denmark found a prevalence of 17.9% among adolescents aged 9 to 16, whereas in France, the rates were 19.6% for children aged 0 to 9 and 42.7% for those aged 10 to 19 [4]. In stark contrast, East Asia exhibits much higher myopia rates, reaching up to 73%. Particularly in East and Southeast Asian countries such as China, Japan, Singapore, and South Korea, nearly 90% of adolescents are myopic by age 18, with high myopia rates approaching 20% [5,6]. Given the serious complications and societal costs associated with myopia, such as myopic choroidal neovascularization, maculopathy, retinal detachment, and glaucoma, it has become a significant clinical and public health issue worldwide [7–9]. These complications not only impact individual health and public health systems but also affect personal quality of life, result in productivity losses, and hinder socioeconomic development [10,11].

In China, the situation among children and adolescents is particularly severe. In 2005, the myopia rate among 15-year-old students was 69.0%, rising to 79.6% in 18-year-olds [12]. Research in China’s western provinces from 2015 indicated an overall adolescent myopia rate of nearly 60% [13]. Between 2020 and 2022, the myopia rate among Chinese high school students reached 83.0%. Thus, China currently has one of the highest myopia prevalence rates globally. This may be partly attributable to genetic factors, as Liu et al. [14] and Li et al. [15] have confirmed the existence of susceptibility genes for myopia [14]. However, related researches also indicate that environmental factors, such as excessive academic workload and inadequate outdoor activities, are closely related to the development of myopia among Chinese children and adolescents [16,17]. Besides, there are significant differences in myopia and high myopia rates among provinces. For example, provinces such as Guangxi and Hainan have lower prevalence rates, while Jiangsu, Shandong, Anhui, and Shanghai have higher rates [18]. In 2022, Chen et al. conducted a study involving cycloplegic refraction on 7,948 students, revealing a myopia prevalence rate of 59.55% and a high myopia prevalence rate of 9.89% in Liyang, Jiangsu Province [19]. In a rural Taiwan study including 6,069 students aged 6 to 15, the myopia rate was 76.6% with high myopia at 2.2% [20]. A 2021 study by Li et al. involving 31,524 students aged 6 to 15 in Hainan reported a myopia rate of 46%, with high myopia at 1% [21]. These differences highlight the importance of targeted regional myopia research for effective prevention and control.

Extensive studies in diverse populations have consistently identified key environmental and behavioural risk factors for myopia development, including limited outdoor time, excessive near work, high academic pressure and increased digital screen exposure [22,23]. Given the strong association between these modifiable factors and myopia, targeted, evidence-based ­interventions have proven effective in reducing myopia ­incidence and progression. For example, a cluster-randomized trial in Guangzhou, China, demonstrated that increasing outdoor activity during school hours significantly lowered myopia onset among primary school children over three years [24], while a population-based study in Australia found an inverse relationship between outdoor time and myopia prevalence in children [25]. Furthermore, the surge in digital screen use during the COVID-19 pandemic correlated with a marked increase in myopia rates, emphasizing the need for early behavioural interventions and screen time regulation [26]. Collectively, these findings underscore the importance of epidemiological investigations to delineate region-specific risk factors and inform tailored myopia prevention strategies.

Currently, there is limited research on the prevalence of myopia among children and adolescents in Shaanxi Province, and there is a particular lack of investigation into the regional differences in myopia prevalence within the province. Shaanxi has a large and diverse population distributed across distinct geographical regions characterized by considerable variations in topography, climate, economic development, and educational resources. These disparities likely lead to differences in environmental exposures and educational pressures, which may influence the risk and prevalence of myopia among children and adolescents [27,28]. Therefore, we conducted an epidemiological study on myopia among school-aged children and adolescents in Shaanxi Province, encompassing the regions of Southern Shaanxi, Guanzhong, and Northern Shaanxi, to investigate potential contributing factors to myopia. This comprehensive study aims to provide valuable insights into the myopia prevalence in different regions of Shaanxi and identify significant risk factors associated with myopia development.

## Methods

### Study design and population

This large-scale, cross-sectional study was conducted in the spring semester of 2021 across Shaanxi Province and included 261,504 children and adolescents aged 6 to 20 years from Guanzhong, Southern Shaanxi, and Northern Shaanxi. The study was approved by the ethics committee of Xi’an People’s Hospital (Xi’an Fourth Hospital) (Approval No. 20210086) and adhered to the principles of the Declaration of Helsinki. Written informed consent was obtained from the parents or guardians of all participants, and the purpose and methods of the study were explained in detail prior to recruitment. A multistage stratified cluster sampling method was used. The sampling procedure involved regional, urban–rural, and school-level randomization steps to ensure representative coverage across Shaanxi Province. A detailed flowchart of the sampling framework is presented in Supplementary Figure 1.

Inclusion criteria comprised school-enrolled children and adolescents aged 6 to 20 years who were able to undergo standard ophthalmic assessments. Exclusion criteria included a history of ocular surgery, presence of ocular diseases (such as amblyopia, strabismus, or congenital anomalies), systemic diseases affecting vision, prior use of myopia correction methods (e.g. orthokeratology or refractive surgery), and inability to complete the examination procedures. Ophthalmic examinations were conducted in classrooms to provide a consistent environment. To standardize educational levels, participants were categorized into Kindergarten, Primary school, Junior High school, and Senior High school, in accordance with the educational structure of Shaanxi Province.

### Ophthalmic examination and questionnaire

Refractive error was measured using a non-cycloplegic auto-refractor (KR-800; Topcon Co., Tokyo, Japan) across all survey sites to ensure consistency. All ophthalmic examinations were conducted by trained optometrists or ophthalmic technicians who underwent standardized training sessions prior to the study, following a unified operating protocol. Each eye was measured three times, and if the discrepancy between any two readings exceeded 0.50 diopters, additional measurements were performed. The mean value of the final consistent measurements was recorded. In cases of uncertainty or technical difficulty, a senior examiner reviewed the results to resolve discrepancies, thereby ensuring the reliability and reproducibility of data across all regions. Spherical equivalent (SE) was calculated as the spherical refractive error plus half the cylindrical refractive error (SE = Sphere + 1/2 Cylinder). Myopia was defined as spherical equivalent (SE) ≤−0.50 diopters (D) and categorized into three grades: low myopia (−0.50 D ≤ SE <−3.00 D), moderate myopia (−3.00 D ≤ SE <−6.00 D), and high myopia (SE ≤ −6.00 D). Although refractive error was measured in both eyes, only the more myopic eye was included in the analyses to avoid inter-eye correlation and to ensure consistency. A structured questionnaire, administered face-to-face by trained researchers, collected demographic information (age, gender, education level, ethnicity) and potential risk factors during ophthalmic examinations.

### Statistical analysis

Continuous variables were expressed as mean ± standard deviation (SD) and categorical variables as frequencies. Myopia prevalence was reported with 95% confidence intervals (CIs). Independent t-tests and R × C chi-square tests were employed to assess group differences, while Spearman correlation analysis explored relationships between SE, age and education level. Age was centered to address multicollinearity with education level in regression models. Univariate and multivariate logistic regression analyses identified risk factors for myopia categories (low, moderate, high). Odds ratios (OR) and 95% CIs were reported, with *p* < 0.05 considered statistically significant.

## Results

### Population demographics

**Figure 1.**
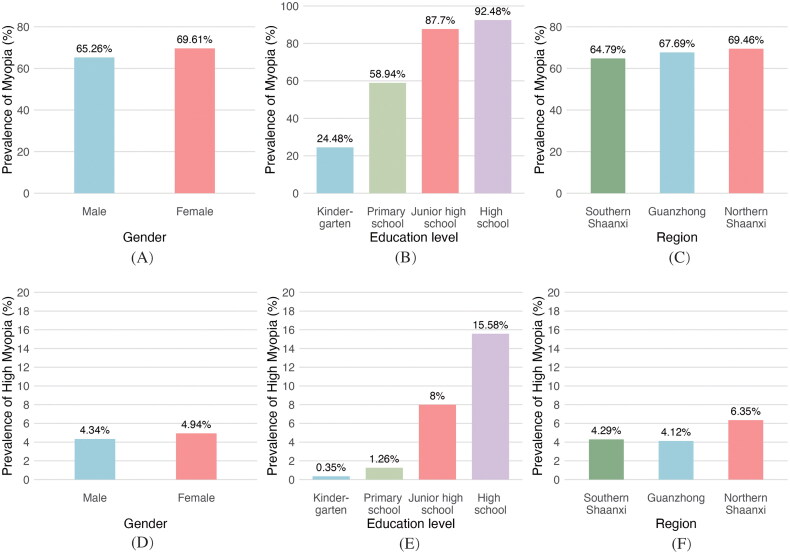
Prevalence of myopia and high myopia among children and adolescents in Shaanxi Province, 2021. Panels (A–C) show myopia prevalence stratified by gender, education level, and region, respectively. Female students demonstrated a higher prevalence than males, with prevalence increasing progressively across education levels. Northern Shaanxi exhibited the highest prevalence compared to other regions. Panels (D–F) illustrate high myopia prevalence by the same categories, showing similar patterns with marked regional differences.

In 2021, a stratified sampling study was conducted across Shaanxi Province, including 261,504 children and adolescents aged 6 to 20 years from Guanzhong, Southern Shaanxi, and Northern Shaanxi. The average age was 14.29 ± 3.47 years, with 51.7% males and 48.3% females. In Southern Shaanxi, the study covered Hanzhong, Ankang, and Shangluo, surveying 62,610 individuals. Of these, 40,562 were myopic (myopia rate: 64.79%), and 2,683 had high myopia (4.29%). In Guanzhong, covering Xi’an, Baoji, Xianyang, and Weinan, 144,129 individuals were surveyed. Myopia prevalence was 67.69% (97,560 myopic cases), with 5,938 high myopia cases (4.12%). Myopia rates were slightly higher in Guanzhong than in Southern Shaanxi, though high myopia was less common. In Northern Shaanxi, the study covered Yan’an and Yulin, with 54,765 participants. Myopia prevalence was 69.46% (38,041 myopic cases), with 6.35% (3,479 cases) having high myopia, the highest among the three regions (Table 1 and Figure 1).

**Table 1.** Epidemiological overview of myopia among children and adolescents across shaanxi Province in 2021.

	[ALL]	Myopia	Non-myopia	*Overall P-value*	χ^2^
	*N = 261504*	*N = 176162*	*N = 85342*		
Sex:				<0.001	561.52
Male	135113	88179	46934		
Female	126391	87983	38408		
Ethnic groups:				<0.001	37.89
Han	246921	165999	80922		
Non-han	14583	10163	4420		
Ages:				<0.001	53175
6–12 years	80153	30787	49366		
13–18 years	133468	101378	32090		
>18 years	47883	43997	3886		
Education level:				<0.001	40669.98
Kindergarten	17808	4359	13449		
Primary school	151823	89584	62239		
Junior High school	55282	48482	6800		
Senior High school	36591	33849	2742		
Region:				<0.001	306.14
Southern Shaanxi	62610	40562	22049		
Guanzhong	144129	97560	46570		
Northern Shaanxi	54765	38041	16724		

### Prevalence of myopia and high myopia

The epidemiological characteristics of myopia in children and adolescents from Shaanxi Province revealed significant gender and grade differences. The overall myopia prevalence was 67.4% (95% CI: 67.20%–67.50%), and high myopia prevalence was 4.63% (95% CI: 4.55%–4.71%). Females had significantly higher myopia (χ^2^ = 561.52, *p* < 0.001) and high myopia rates (χ^2^ = 53.123, *p* < 0.001) than males. Myopia prevalence increased progressively from kindergarten to high school. Myopia prevalence increased consistently with educational progression. Compared with kindergarten students (24.48%), the prevalence was markedly higher in primary school (58.94%), junior high school (87.70%), and peaked in senior high school (92.48%). A similar trend was noted for high myopia, with prevalence rising from 1.26% in primary school to 8.00% in junior high school and 15.58% in senior high school, representing a nearly 12-fold increase. Regionally, Northern Shaanxi had the highest myopia prevalence (69.46%, 95% CI: 69.1%–69.8%) and high myopia prevalence (6.35%, 95% CI: 6.15%–6.56%) compared to Southern Shaanxi and Guanzhong (Table 2).

**Table 2.** Myopia and high myopia prevalence among children and adolescents in shaanxi, 2021.

	[ALL] /n	Myopia rate (95% CI), %	*Overall P-value*	χ^2^		[ALL] /n	High myopia rate (95% CI), %	*Overall P-value*	χ^2^
Myopia					High myopia				
Totality	176162	67.40 (67.20–67.50)	<0.001	561.44		12102	4.63 (4.55–4.71)	<0.001	53.123
Male	88179	65.30 (65.00–65.50)				5861	4.34 (4.23–4.45)		
Female	87983	69.60 (69.40 − 69.90)				6241	4.94 (4.82–5.06)		
Education level:			0.000	40701.71				<0.001	15990
Kindergarten	17808	24.50 (23.80 − 25.10)				63	0.35 (0.28–0.45)		
Primary school	151823	58.90 (58.70–59.20)				1919	1.26 (1.21–1.32)		
Junior High school	55282	87.70 (87.40–88.00)				4420	8.00 (7.77–8.22)		
Senior High school	36591	92.50 (92.20 − 92.80)				5700	15.6 (15.2–16.0)		
Region:			<0.001	306.14				<0.001	469.53
Southern Shaanxi	62610	64.80 (64.40-–65.20)				2683	4.29 (4.13–4.45)		
Guanzhong	144130	67.7 (67.4–67.9)				5938	4.12 (4.02–4.23)		
Northern Shaanxi	54765	69.50 (69.10–69.80)				3479	6.35 (6.15–6.56)		

### Changes in spherical equivalent (SE) across regions among myopic children and adolescents

**Figure 2.**
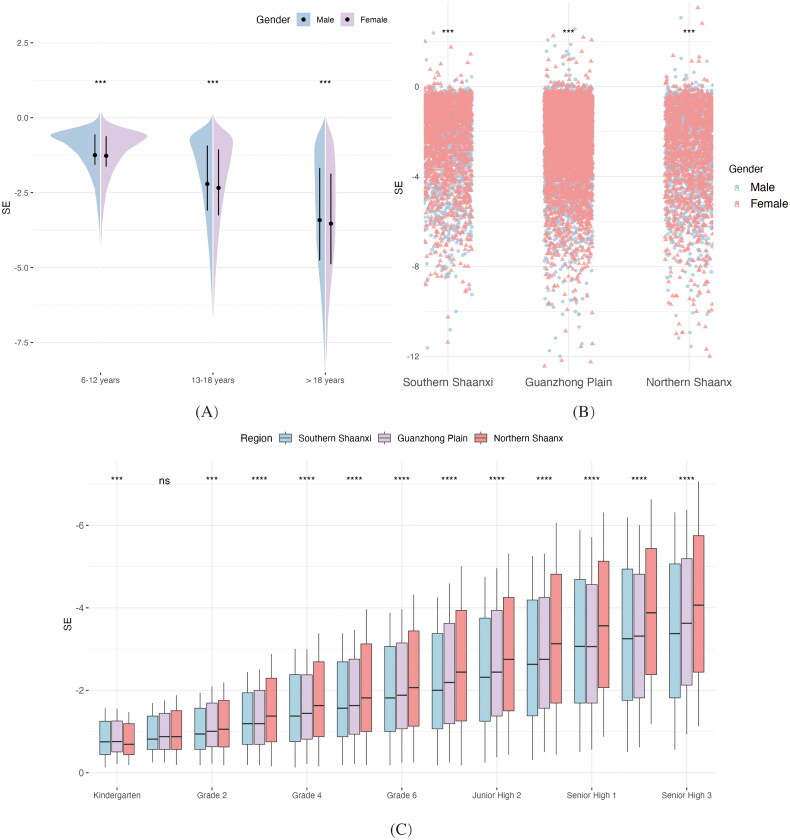
Regional differences in spherical equivalent (SE) among myopic children and adolescents in Shaanxi Province, 2021. Panel (A) depicts SE distributions by age and gender, showing increasing myopic severity with age and generally greater myopia in females. Panel (B) presents SE values by region and gender, highlighting more severe myopia in Northern Shaanxi, particularly among females. Panel (C) shows SE variation by educational grade across regions, with a progressive myopic shift evident, especially in Northern Shaanxi. The average SE of both eyes was used. Abbreviations: SE, spherical equivalent; D, diopters. Significance: ***p < 0.001, **p < 0.01, *p < 0.05, ns = not significant.

Spherical equivalent variations in myopic children and adolescents from Shaanxi Province were comprehensively analysed. Figure 2A demonstrates age- and gender-specific SE variations, revealing a progressive increase in myopic refractive error with advancing age. Across all age groups, females exhibited more severe myopia than males, with the difference becoming more pronounced with age. For example, at ages 6–12, the average SE for myopic males and females were −1.25 ± 1.06D and −1.27 ± 1.07D, respectively. Among individuals aged 13–18, females showed a significantly greater myopia severity, with SE values of −2.34 ± 1.71D versus −2.21 ± 1.67D (*p* < 0.001). The gender gap widened further in those over 18, with females showing a 0.11D greater myopia (*p* < 0.001). Regional comparisons revealed that females exhibited higher myopia severity than males in all regions, with the most pronounced difference in Northern Shaanxi, where females’ average SE was 0.20D lower than males’ (*p* < 0.001). (Figure 2B).

Grade-specific SE distributions were analysed for myopic individuals in each region (Figure 2C). SE values progressively became more negative with higher educational levels, indicating greater myopia severity. Among students in Southern Shaanxi, the most pronounced SE change occurred between Senior High 2 and Senior High 3, with an average increase of 0.09D in absolute SE. In Guanzhong and Northern Shaanxi, the largest SE change occurred between the first and second grade of primary school, with Northern Shaanxi students showing an average increase of 0.16D in absolute SE. Northern Shaanxi students had the highest absolute SE across all grades (−2.99 ± 2.02D), with Senior High 3 students showing a 0.56D higher SE than those in Southern Shaanxi (*p* < 0.001).

### Analysis of myopia risk factors in Shaanxi province

Based on the present dataset, risk factors for myopia among children and adolescents in Shaanxi Province were systematically investigated. Correlation analysis demonstrated a significant negative association between myopia severity in both eyes and factors such as age and educational attainment (Supplementary Figure 2). Subsequent univariable and multivariable regression analyses were conducted to evaluate the overarching risk factors for myopia. The findings indicated that gender, grade level, and geographic region were significant determinants of myopia risk. In univariable regression analysis, females exhibited a markedly higher risk of myopia compared to males (OR = 1.22; 95% CI: 1.20–1.24). This association was reinforced in the multivariable analysis, which revealed an even greater risk for females (OR = 1.27; 95% CI: 1.25–1.29). Regionally, the risk of myopia was significantly elevated in Guanzhong (OR = 1.14; 95% CI: 1.12–1.16) and Northern Shaanxi (OR = 1.24; 95% CI: 1.21–1.27) compared to Southern Shaanxi in univariable analysis. Multivariable regression confirmed these regional disparities, underscoring their persistence even after adjusting for gender and grade level. Furthermore, the risk of myopia increased progressively with advancing grade levels. In univariable analysis, the risk rose from the first grade of primary school to the third grade of high school, culminating in an OR of 48.56 (95% CI: 44.04–53.53). This trend was consistent in multivariable analysis, with a peak OR of 49.21 (95% CI: 44.63–54.26), highlighting the compounded impact of grade level when accounting for gender and region (Table 3).

**Table 3.** Univariate and multivariate regression analysis of myopia risk in children and adolescents in Shaanxi Province.

Dependent: Visual status		Myopia (*N* = 176163)	Univariable analysis			Multivariable analysis		
			OR	95% CI	*P-value*	OR	95% CI	*P-value*
Sex	Male	88179 (50.10%)						
	Female	87984 (49.90%)	1.22	1.20–1.24	<0.001	1.27	1.25–1.29	<0.001
Area	Southern Shaanxi	40562 (230%)						
	Guanzhong	97560 (55.40%)	1.14	1.12–1.16	<0.001	1.27	1.24–1.30	<0.001
	Northern Shaanxi	38041 (21.60%)	1.24	1.21–1.27	<0.001	1.28	1.24–1.32	<0.001
Grade	Kindergarten	4359 (2.50%)						
Primary School	Grade 1	8903 (5.10%)	1.55	1.48–1.61	<0.001	1.54	1.47–1.60	<0.001
	Grade 2	12080 (6.90%)	2.58	2.48–2.69	<0.001	2.57	2.46–2.68	<0.001
	Grade 3	15283 (8.70%)	4.26	4.08–4.44	<0.001	4.25	4.07–4.43	<0.001
	Grade 4	17330 (9.80%)	6.38	6.11–6.66	<0.001	6.38	6.11–6.66	<0.001
	Grade 5	17784 (10.10%)	8.71	8.33–9.11	<0.001	8.73	8.35–9.13	<0.001
	Grade 6	18102 (10.30%)	12.70	12.11–13.32	<0.001	12.81	12.21–13.43	<0.001
Junior High School	7th grade	16402 (9.30%)	16.91	16.06–17.81	<0.001	17.15	16.28–18.07	<0.001
	8th grade	16727 (9.50%)	22.85	21.61–24.15	<0.001	23.19	21.93–24.52	<0.001
	9th grade	14854 (8.40%)	30.23	28.39–32.20	<0.001	30.76	28.88–32.76	<0.001
Senior High School	10th grade	13619 (7.70%)	36.76	34.30–39.41	<0.001	36.86	34.38–39.51	<0.001
	11th grade	13025 (7.40%)	34.89	32.55–37.39	<0.001	35.05	32.70–37.57	<0.001
	12th grade	7695 (4.40%)	48.56	44.04–53.53	<0.001	49.21	44.63–54.26	<0.001

OR, odds ratio; CI, confidence interval; SE, spherical equivalent. Odds ratios and 95% confidence intervals are presented for univariable and multivariable analyses. The multivariable models were adjusted for gender, region (southern Shaanxi, Guanzhong, and northern Shaanxi), and grade level (Kindergarten to Senior High School).

The associations between gender, educational level, and varying degrees of myopia (low, moderate, and high) were assessed across multiple regions-Southern Shaanxi, Guanzhong, and Northern Shaanxi-to elucidate regional differences. Both univariable and multivariable analyses consistently indicated a higher myopia risk for females in all regions and across all severity levels. For example, the multivariable analysis for high myopia revealed an OR of 1.57 (95% CI: 1.49–1.66) for females in Guanzhong, with Northern Shaanxi demonstrating an even greater risk (OR = 1.78; 95% CI: 1.60–1.99). The impact of grade level on myopia prevalence intensified with increasing severity. In Guanzhong, univariable analysis showed ORs of 1.34 (95% CI: 1.33–1.34) for low myopia, 1.69 (95% CI: 1.68–1.70) for moderate myopia, and 1.79 (95% CI: 1.77–1.80) for high myopia. Multivariable regression confirmed the independent effects of gender and grade level on myopia risk, even after accounting for confounding variables.

The regional differences in myopia risk were also pronounced. In multivariable analysis, the OR for low myopia was lowest in Guanzhong (OR = 1.18; 95% CI: 1.16–1.21) and highest in Southern Shaanxi (OR = 1.31; 95% CI: 1.26–1.36). For moderate myopia, Southern Shaanxi and Northern Shaanxi exhibited higher risks (OR = 1.50; 95% CI: 1.41–1.59 and OR = 1.62; 95% CI: 1.52–1.72, respectively). High myopia risk was greatest in Northern Shaanxi (OR = 1.78; 95% CI: 1.60–1.99), significantly surpassing that in Guanzhong and Southern Shaanxi (Table 4).

**Table 4.** Impact of gender and education on various degrees of myopia across regions in shaanxi Province.

Dependent: Visual status		Myopia	Univariable analysis			Multivariable analysis		
			OR	95% CI	*P*-value	OR	95% CI	*P*-value
**Southern Shaanxi**								
**Mild myopia**								
Sex	Male	12951 (49.10%)						
	Female	13409 (50.90%)	1.25	1.20–1.29	<0.001	1.31	1.26–1.36	<0.001
Grade	Mean ± SD	6.6 ± 3.1	1.32	1.31–1.33	<0.001	1.32	1.31–1.33	<0.001
**Moderate myopia**								
Sex	Male	5389 (46.80%)						
	Female	6130 (53.20%)	1.37	1.31–1.43	<0.001	1.50	1.41–1.59	<0.001
Grade	Mean ± SD	8.9 ± 2.7	1.66	1.64–1.67	<0.001	1.66	1.64–1.68	<0.001
**High myopia**								
Sex	Male	1318 (49.10%)						
	Female	1365 (50.90%)	1.25	1.15–1.35	<0.001	1.45	1.31–1.61	<0.001
Grade	Mean ± SD	10.0 ± 2.5	1.80	1.77–1.83	<0.001	1.80	1.77–1.84	<0.001
**Guanzhong**								
**Mild myopia**								
Sex	Male	55134 (51%)						
	Female	52866 (49%)	1.13	1.11–1.15	<0.001	1.18	1.16–1.21	<0.001
Grade	Mean ± SD	5.9 ± 2.9	1.34	1.33–1.34	<0.001	1.34	1.34–1.35	<0.001
**Moderate myopia**								
Sex	Male	21358 (49%)						
	Female	22239 (51%)	1.23	1.20–1.26	<0.001	1.38	1.34–1.42	<0.001
Grade	Mean ± SD	8.3 ± 2.8	1.69	1.68–1.70	<0.001	1.69	1.68–1.70	<0.001
**High myopia**								
Sex	Male	4389 (47.60%)						
	Female	4831 (52.40%)	1.30	1.24–1.35	<0.001	1.57	1.49–1.66	<0.001
Grade	Mean ± SD	9.4 ± 2.7	1.79	1.77–1.80	<0.001	1.79	1.77–1.81	<0.001
**Northern Shaanxi**								
**Mild myopia**								
Sex	Male	11690 (53.50%)						
	Female	10167 (46.50%)	1.16	1.11–1.21	<0.001	1.23	1.17–1.28	<0.001
Grade	Mean ± SD	6.3 ± 3.1	1.34	1.33–1.35	<0.001	1.34	1.33–1.35	<0.001
**Moderate myopia**								
Sex	Male	6165 (48.50%)						
	Female	6540 (51.50%)	1.42	1.35–1.48	<0.001	1.62	1.52–1.72	<0.001
Grade	Mean ± SD	8.7 ± 2.7	1.73	1.71–1.75	<0.001	1.73	1.71–1.75	<0.001
**High myopia**								
Sex	Male	1679 (48.30%)						
	Female	1800 (51.70%)	1.43	1.33–1.54	<0.001	1.78	1.60–1.99	<0.001
Grade	Mean ± SD	10.1 ± 2.4	1.97	1.93–2.01	<0.001	1.97	1.93–2.02	<0.001

OR, odds ratio; CI, confidence interval; SE, spherical equivalent; D, diopters.

#1: this model was performed with ‘mild-myopia (SE: <−0.5 to >−3.0 D)’ and ‘non-mild-myopia (SE: >−0.5 and <−3.0 D)’ as dependent variable.

#2: this model was performed with ‘moderate-myopia (SE: <−3.0 to >−6.0 D)’ and ‘non-moderate-myopia (SE: >−3.0 and <−6.0 D)’ as dependent variable.

#3: this model was performed with ‘high-myopia (SE: <−6.0 D)’ and ‘non-high-myopia (SE: >−6.0 D)’ as dependent variable.

The multivariable models were adjusted for gender, grade, and region (southern, Guanzhong, and northern Shaanxi).

These findings provide critical insights into the demographic and regional disparities influencing myopia development and progression, emphasizing the importance of targeted preventive strategies tailored to the unique needs of each subgroup.

## Discussion

In 2021, the overall myopia prevalence in Shaanxi Province was 67.4%, and high myopia prevalence was 4.6%. Compared to other countries, Lundberg’s 2018 study found a myopia rate of 17.9% among Danish adolescents aged 9–16 [29]; in France, myopia rates were 19.6% for children aged 0–9 and 42.7% for adolescents aged 10–19 [30]. The Irish Eye Study, which surveyed 1,626 participants, found that the prevalence of myopia was 3.3% among children aged 6–7 years and 19.9% among those aged 12–13 years [31]. A German study found that myopia prevalence in children and adolescents aged 0–17 remained stable at around 11.6% over approximately 10 years [6]. The development of myopia is even more severe in East Asia. For example, in South Korea, myopia prevalence reached 73% among students, with high myopia at 5%, based on the 2008-2012 Korea National Health and Nutrition Examination Survey [32]. In 2019, Yotsukura et al. found a myopia rate of 76.5% among Japanese elementary students aged 6–14, with high myopia at 4%; high school myopia prevalence increased to 94.9%, with high myopia at 11.3% [33]. Thus, the myopia and high myopia rates in Shaanxi Province are relatively high globally and within Asia. Comparatively, in other Chinese provinces, a 2021 study by Li et al. reported a myopia rate of 46% among 31,524 students aged 6–15 in Hainan, with high myopia at 1% [21]. In 2019, myopia prevalence among Chengdu students aged 3–14 was 38.1%, with high myopia at 1.7% [34]. However, in 2023, a study in Shenyang, Liaoning Province, examined 34,644 students with a median age of 11.9 years, revealing a significantly higher myopia prevalence of 60% and a high myopia prevalence of 1.9% [35]. Thus, Shaanxi Province’s myopia and high myopia rates are also high within China, surpassing the domestic average of 52.7% [36]. Compared to the 2018 data for Shaanxi Province, when the myopia detection rate among children and adolescents aged 5–18 in Shaanxi was 54.9%, with equal rates for boys and girls at 58.6% [37], the 2021 study showed a significant increase in myopia rates for both boys and girls [38].

Myopia rates have shown a clear upward trend across all educational stages, rising from below one-quarter in kindergarten to over 90% in high school, where more than one in seven students are already affected by high myopia. Despite policies aimed at reducing school workload, the burden remains substantial [39]. The rising use of electronic devices, particularly for studying, gaming, and social media, contributes to myopia by increasing near work, promoting axial elongation, and reducing outdoor activity, which is protective against myopia [5,23]. Notably, the COVID-19 pandemic worsened this trend, with lockdowns increasing screen time and exacerbating myopia severity [26]. A study in Shanxi found that myopia progression in elementary school students during the pandemic was strongly correlated with screen time and device types [40]. Therefore, Strategies to limit screen time and encourage outdoor activities are essential for curbing the growing myopia prevalence. Regional analyses highlighted notable geographic variations in both myopia and high myopia prevalence across Shaanxi’s three regions (Table 2). Northern Shaanxi had the highest rates and SE values among myopic students, possibly linked to dietary factors like a wheat- and meat-based diet and insufficient vitamin D intake [41]. Myopia rates have notably increased in all regions over the past three years (Southern Shaanxi: 51.7%; Guanzhong: 54.7%; Northern Shaanxi: 58.7%) [37].

Further analysis revealed that female gender is significantly associated with a higher prevalence of both myopia and high myopia, which may be attributed to a combination of cultural and physiological factors. In China, girls engage more in indoor activities, resulting in longer near-work durations, and puberty is independently linked to myopia in females, unlike in males [42]. Mutations in X-linked cone opsin genes are associated with myopia development. Girls, with two X chromosomes, are twice as likely as boys to carry polymorphic variants [43]. The conclusions align with research from other countries like Finland [44] and Poland [45], indicating that sex differences in ophthalmic and physiological optics are influenced by genetic factors, dietary habits, amount of near work, and puberty. Distinct regional gender disparities in myopia were observed, particularly in Southern Shaanxi for moderate myopia and in Guanzhong for high myopia (Table 3). Further investigation into these geographic variations is needed.

Moreover, higher education levels correlated with increased myopia and high myopia rates, consistent with findings from multiple studies worldwide [46,47]. Higher education levels lead to more indoor near-work time and less outdoor activity time. Specifically, higher levels of education involve increased study efforts, such as hours of homework per day on weekends, the frequency of after-school tutoring per week, and engagement in extracurricular reading, all significantly associated with the development and progression of myopia [48]. In this study, grade level was a significant factor influencing myopia risk, particularly in key transition years such as sixth, ninth, and twelfth grades within the Chinese education system. Overall, these findings underscore the urgent need for targeted public health policies and interventions to curb myopia progression.

Despite the study’s strengths, including a large sample size and comprehensive coverage, some limitations should be noted. Firstly, as a cross-sectional study conducted in 2021, it identifies associations between factors such as gender, region, and grade with myopia prevalence but cannot establish causality. Future longitudinal studies are required to clarify whether these associations are causal. Secondly, the findings are specific to Shaanxi Province and may not be generalizable to other regions or populations due to differences in diet, climate, and educational practices. Caution is advised when extrapolating these results to areas with distinct socio-economic or environmental conditions. Thirdly, non-cycloplegic autorefraction was utilized owing to its practicality and suitability for large-scale epidemiological investigations, facilitating efficient data collection with minimal participant burden. While non-cycloplegic refraction is not regarded as the gold standard for refractive error assessment—cycloplegic refraction remains the criterion standard, particularly for younger children with high accommodative tone—it offers a feasible alternative in large population-based studies. The potential overestimation of myopia prevalence, especially among younger age groups, is acknowledged; nonetheless, the stability of observed associations across subgroups and the limited influence on estimates of high myopia prevalence support the validity and reliability of the principal findings. Lastly, the absence of detailed information on daily visual habits, learning environments, and physical activity constitutes a limitation, as it restricts a more comprehensive assessment of potential risk factors. This limitation primarily stems from the practical constraints associated with collecting extensive behavioural data in a province-wide epidemiological survey of this scale, encompassing a large and diverse student population. Future investigations should incorporate more detailed and targeted data collection to better elucidate the multifactorial aetiology of myopia.

This study provides a detailed analysis of myopia prevalence and associated factors among school-aged children and adolescents in Shaanxi Province. It highlights increasing trends in myopia and high myopia prevalence and the relationships with gender, region, and grade. Based on the present findings, we emphasize the necessity for comprehensive public health strategies that incorporate early identification and management of myopia, targeted educational interventions focused on vulnerable subpopulations, and environmental modifications conducive to ocular health. Additionally, addressing region-specific risk factors, including potential nutritional deficiencies and lifestyle determinants, is critical for the effective mitigation of myopia prevalence. These integrated approaches should inform policy frameworks aimed at reducing the burden of myopia and its associated complications in school-aged populations.

## Supplementary Material

supplementation figure1.tif

supple figure2.tif

## Data Availability

The data that support the findings of this study are available from the corresponding author upon reasonable request.
