# Supplementary figures and images for: Cross-sectional study of myopia prevalence and associated risk factors among children and adolescents in Shaanxi Province, China, in 2021
Source: Ann Med. 2025 Jun 22;57(1):2522319. doi: 10.1080/07853890.2025.2522319 (PMC12931328; doi:10.1080/07853890.2025.2522319)

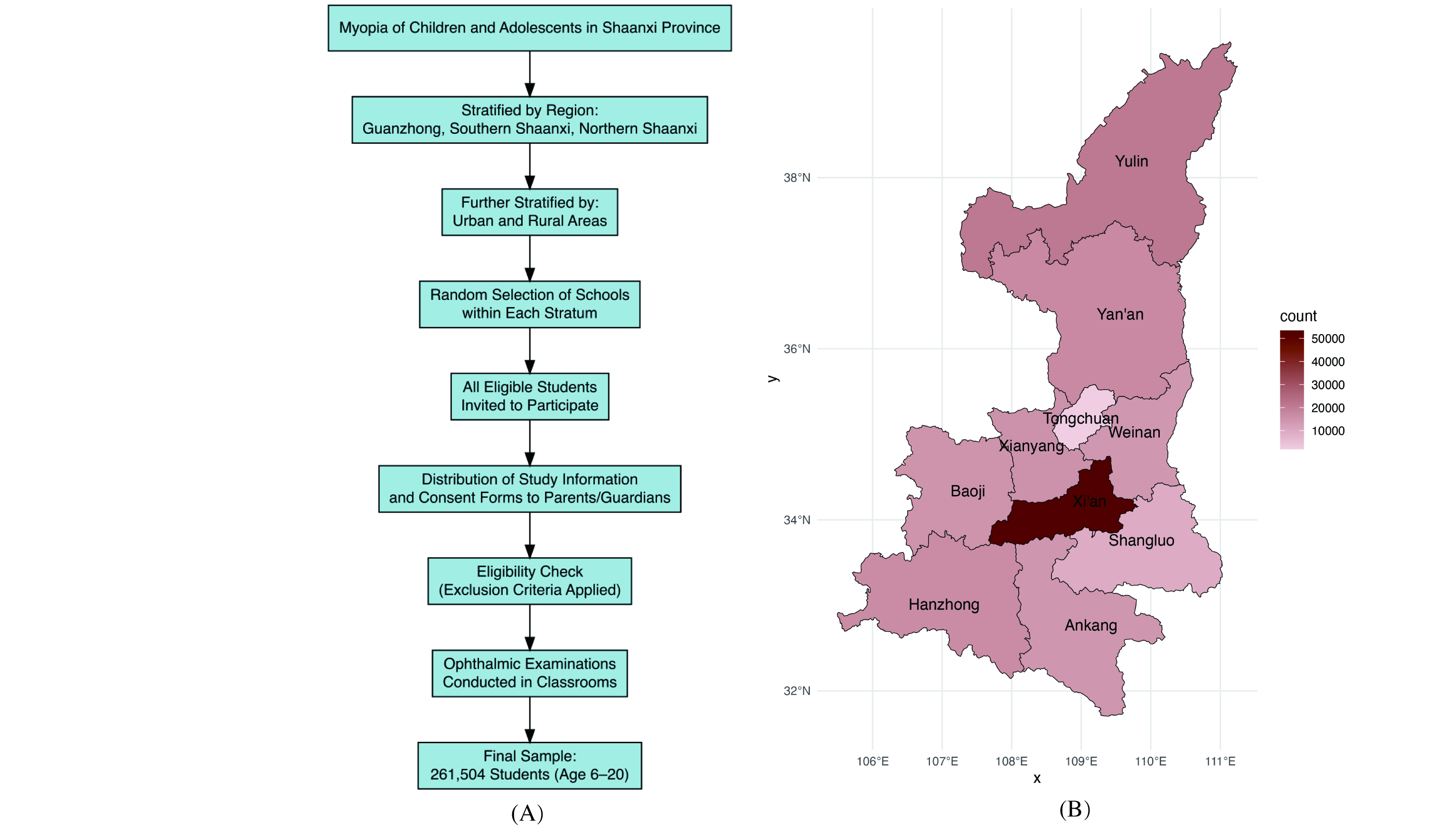

Supplement: supplementation figure1.tif [file IANN_A_2522319_SM8099.tif]

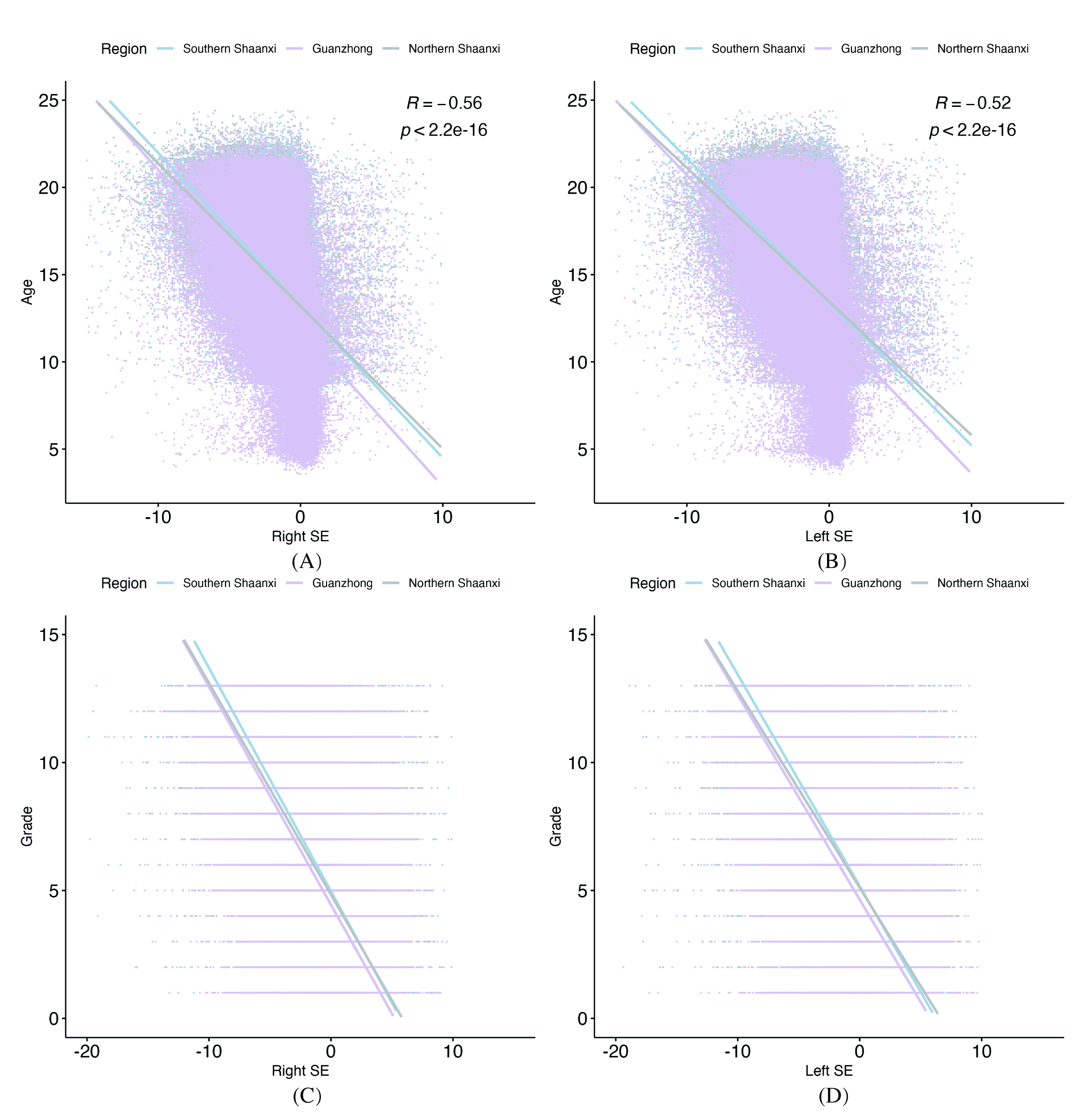

Supplement: supple figure2.tif [file IANN_A_2522319_SM8098.tif]
